# Perceived post-migration discrimination: the perspective of adolescents with migration background

**DOI:** 10.1007/s00787-022-02084-6

**Published:** 2022-09-21

**Authors:** Andrea Borho, Eva Morawa, Caterina Schug, Yesim Erim

**Affiliations:** Department of Psychosomatic Medicine and Psychotherapy, University Hospital Erlangen, Friedrich-Alexander University Erlangen-Nürnberg (FAU), Schwabachanlage 6, 91054 Erlangen, Germany

**Keywords:** Discrimination, Refugees, Migrants, Adolescents, Youth

## Abstract

Discrimination has a negative impact on mental health and wellbeing of persons affected. The aim of the present study was to investigate perceived discrimination of migrant adolescents. Focus groups with migrant adolescents were conducted, audio-recorded and transcribed verbatim. The transcripts were analyzed using the manifest qualitative content analysis by Mayring. The foci of interest (persons discriminated against and by whom, where and how the discrimination took place, which behavioral and emotional reactions the affected persons showed to the experiences and which reasons they assumed for the discrimination) were determined based on the pre-defined guideline, and inductive subcategories were extracted from the transcripts and grouped into main categories. Five focus groups with students with migration background (*n* = 35, 13 females, mean age: 18.78, SD = 2.26, range 16–24) were conducted. The adolescents in our focus groups and their acquaintances (families and friends) experienced discrimination in Germany in several forms (verbal and non-verbal), places and on an individual as well as institutional level, with an impact on their emotional well-being. To combat discrimination, culturally sensitive information must be provided on the part of those affected as well as their perpetrators.

## Introduction

Migration is a global phenomenon and has occurred in all nations at all times [1]. The process of migration itself is extremely heterogeneous, can be forced or voluntary, and poses a great burden on mental health. In addition to pre-migration stressors in countries of origin (such as armed conflicts, socioeconomic crises, etc.), adaptation to a new environment (post-migration) also includes potential socioeconomic, social, interpersonal, and migration-related stressors (as adverse social circumstances, low socioeconomic status, work and school difficulties, bilingualism, etc.), that have been associated with impaired psychological functioning and poorer mental health [1–4].

Among these post-migration stress factors, discrimination is one of the most burdening, significantly worsening the health and well-being of young people with migration background [5]. Research with adult as well as immigrant and refugee youth underscores the negative impact of discrimination on both physical (e.g., self-rated, hypertension, cardiovascular, respiratory) and mental health (affective disorders, e.g., anxiety, depression) [6–8]. A recent study among 1366 refugee and non-refugee migrant young people in five European countries revealed that perceived discrimination was associated with increased levels of mental health problems and lower overall well-being for refugees and non-refugee migrants. Although non-refugee migrants reported higher levels of perceived discrimination than refugees did, it posed equal risks to the mental health of both groups [5]. For refugees specifically, previous findings provide additional evidence for the relationship between experiences of discrimination and PTSD-symptomatology [9]. Research examining the link between discrimination and health for this population suggests that discrimination may be particularly damaging for refugees and asylum seekers, reactivating traumatic suffering [7, 10]. In addition to directly experiencing discrimination, witnessing or experiencing vicarious discrimination, can also negatively affect mental health [11]. A report by Correa-Velez et al. [12] also proved that the negative impact of discrimination on refugee youth well-being persists over time [12].

Besides its negative impact on health, perceived discrimination seems to play a major role in the development of acculturative stress [13] and hinders the psychological, social, and sociocultural adaption to the host country [14]. It also lowers self-esteem [15], promotes antisocial behavior [16, 17], and it negatively affects the cultural identity and sense of self of immigrant adolescents [18]. Experiences of discrimination can lead to resignation or to behavioral restrictions as well as to sadness, anger or also to aggressions [19]. Beyond these psychological consequences, there are behavioral consequences as well. Migrant youths who perceive greater levels of discrimination are more likely to drink, smoke, and use illegal substances [20]. In the school context, discrimination can lead to poorer school adjustment [21] and engagement [22], particularly when discrimination occurs in the school environment. The results of a Chinese study among 2041 migrant adolescents also indicated that teacher discrimination was positively associated with depression [23].

Discrimination can occur in different forms and areas of life. It can be overt or covert, conscious or sub-conscious and can occur as interpersonal interactions between individuals, as institutionally engrained, systemic practices, policies or processes, or as internalized ideologies, beliefs or attitudes about the inferiority of a person or group [24, 25]. It can range from unfriendly behavior or social exclusion over the refusal of services such as housing, employment or education up to verbal threats and physical hostilities [26]. Discrimination can be based on race, language, religion, country of origin, physical appearance, and/or other aspects that characterize a group or a person as “different” [24, 27].

People with refugee or migration background belong to a group that is particularly often affected by discrimination in working life, in the housing market, in access to goods or services, at agencies and authorities, and within their neighborhoods [27–29]. Moreover, institutional discrimination is evident in government policies, such as restrictions on services for those on temporary visas. Young migrants are also at risk of experiencing discrimination in the context of school and education. In this respect, previous findings indicate that young people with a migration background are at a disadvantage when it comes to accessing higher education and vocational training [30].

In 2018, our team launched a prevention project of mental disorders for refugee students in cooperation with a vocational school in Germany. At the beginning, the project consisted of two parts: psychoeducational lectures on trauma and mental disorders and the assessment of psychosocial distress by means of a screening instrument (Refugee-Health-Screener 15), symptom-specific questionnaires (e.g., Patient-Health-Questionnaire-9 and 15) as well as psychosocial stress factors, such as discrimination (self-prepared questionnaire). After analyzing the results of the study, it was found that 51% of refugee students had already experienced discrimination at school. This high percentage was followed by experiences of discrimination in public offices (47%), in their neighborhoods (38%) and while shopping (32%) (results are not published yet). As the vocational school has been involved in the initiative “School without Racism, School with courage” [31] (an initiative of the association “Aktion Courage e.V.” and funded, among others, by the Federal Ministry for Family Affairs, Senior Citizens, Women and Youth for all school members to actively oppose discrimination, in the form of activities, such as discussions, theater performances, or exhibitions) for several years, the school was very interested in investigating the background of these results in more detail. Since previous studies have proven the strong link between discrimination and mental health issues [5, 7, 8], it was important from both the school and the scientific perspective, to understand which factors play a role in these high percentages. Also with regard to the integration of the currently arriving refugees from Ukraine [32], among them many children and adolescents, these aspects will again be of great importance. Therefore, we designed a qualitative study to explore the specific aspects of perceived discrimination in more detail among a sample of adolescents with migration background in the same vocational school. Following Sawyer et al. [33], we defined the age of 10–24 as adolescence, as it describes best the growth of adolescents and the common understanding of this stage of life [33]. According to the German Federal Statistical Office, migrants were defined as people who were not born with the German citizenship but changed their country of origin, irrespective of the reason for migration or legal status (including flight and voluntary migration) [34].

Including these definitions, the aim of the presented study was to examine the following research questions among a sample of migrant adolescents:

1. Who exactly experiences discrimination and from whom?
2. In which areas of life and in which forms does the discrimination manifest itself?
3. What are the reactions of the persons affected to the experiences of discrimination?
4. What reasons do they assume for these discriminatory experiences?

## Methods

From July 22 to 25, 2019, we conducted a qualitative study consisting of focus groups on experiences of discrimination with migrant students in a vocational school in a big city (> 100,000 residents) in Bavaria (Germany).

### Participants and procedure

We conducted five focus groups with in total 35 vocational students with migration background. The students included (unaccompanied) refugees, as well as voluntary migrants who came to Germany with their parents. All participants went to vocational integration classes that were specifically established for these students. The vocational integration classes in Bavaria are a two-year program funded by the Bavarian Ministry of Education and Cultural Affairs and directed at migrants who are required to attend vocational school (from 16 to 25 years) and who have insufficient/absent knowledge of German language. The general objective of the teaching concept is the teaching and promotion of language skills, vocational orientation and preparation for training, as well as the support for social integration by social pedagogues. In these classes, the young people are supported in gaining qualifications and preparing for their entry into the labor market. At the time of the study, a total of 67 students were attending the five vocational integration classes at the vocational school in question. However, only 35 of them participated (52%). Reasons for non-participation were: absence on the day of the conduction of the focus groups, lack of consent (from parents or guardians), no interest in participating, and desire not to talk about negative events (especially in the case of Ethiopians due to traumatic experiences in their home country). In our study, the 22 male and 13 female participants were on average 18.78 years old (range = 16–24, SD = 2.26). Their countries of origin were Syria, Iraq, Ukraine, Armenia, Afghanistan, Iran, Pakistan, Hungary, Romania, Greece, Albania, Turkey, and Ethiopia.

The sessions lasted on average 40 min (range 20–57 min) and were conducted by two researchers in German language. The discussions were recorded with two audio recording devices. Each group was moderated by a researcher who guided the group discussion, and an observer. All focus groups started with a general question about previous experiences of discrimination in Germany followed by other transition questions to go deeper in the discussion. For all focus groups, we followed a pre-defined semi-structured interview guide consistent of questions such as:

1. Have you experienced situations where you felt treated unfairly?
2. In which area of your life (places) did you have these experiences?
3. How often have you experienced such situations?
4. Why do you think you were treated unequally?
5. By whom were you treated unequally?
6. How did you react to the unequal treatment?
7. What support would you like to have for such situations?
8. How do you think you would have been treated as a German in this situation?

As the groups only included students who were interested in sharing their experiences, the group dynamics were positive throughout. Almost all of the participants discussed the different topics in the interview guide; when some individuals were dominating the discussion, the moderator invited other participants to comment whilst being mindful not to control the discussion but creating space for every participant to contribute. In each group there were more dominant and more shy participants, but each student was given the space to open up and share their experiences. Although the groups were mixed with female and male participants and already knew each other, they seemed to be able to talk openly and without shame. The biggest obstacle in these focus groups was the lack of German language skills of some participants. However, with the help of the moderators and fellow students, they managed to express themselves. Due to the different languages in each focus group, no interpreter could be called in.

### Coding and analysis

The discussions were anonymized and transcribed verbatim to enable deductive and inductive thematic content analysis. Since some of the students still had considerable difficulties with the German language, the wording of the students' statements has been slightly improved for this article for better understanding.

**Fig. 1:**
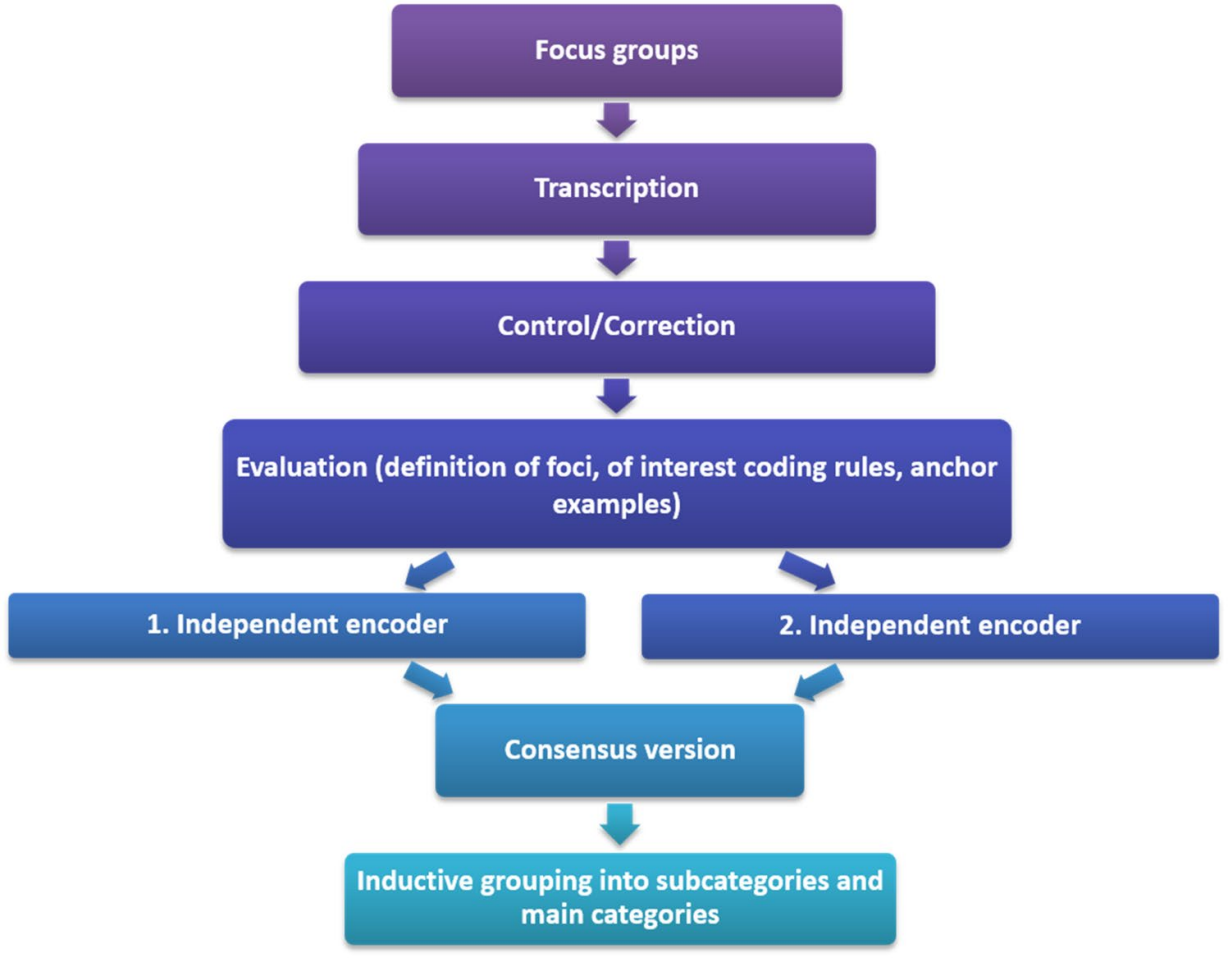
Procedure of coding and analyzing the focus groups

To increase reliability, each transcript was read and coded by two researchers independently, using the software package ATLAS-ti, version 7.5.18 (Atlas.ti Scientific Software Development GmbH, Berlin, Germany). The focus groups were evaluated on the basis of the manifest qualitative content analysis according to Mayring [35]. Accordingly, in a joint discussion among two of the researchers, the following foci of interest were deductively formed based on the research interests: Person(s) who perceived discrimination, discriminating person(s), forms of discrimination, locations where discrimination has taken place, reactions of the person(s) affected to the discrimination, and reasons for discrimination as assumed by the students. For each focus of interest, anchor examples and coding rules were defined. According to the coding rules, every statement (each word/sentence fragment) was coded that has subjectively been perceived as discrimination by the students and could be assigned to one of the foci of interest. As anchor examples, we selected one exemplary code from the interviews for each focus of interest. Following the coding rules, the found statements were marked and assigned to the corresponding foci of interest. In the next step, codes made by the two independent evaluators were checked for consistency, discussed, and revised accordingly until a common consensus was reached. Finally, all statements assigned to one of the foci of interest were inductively divided into thematic subcategories. To quantify the experiences reported by the students, absolute frequencies were given for the subcategories identified. For the focus of interest “Reactions of the person(s) affected to the discrimination” the identified subcategories were summarized under two main categories “Actions” and “Emotions/affects”. The detailed process of coding and analyzing is illustrated in (Fig. 1).

### Ethical considerations

This study was approved by the Ethics Committee of the Medical Faculty of the Friedrich‐Alexander‐University Erlangen‐Nürnberg (FAU) (file reference: 319_17 B). All participants were informed verbally and in written form about the study before signing a written informed consent form. They were aware that participation was voluntary, that their information would be kept confidential and that it would only be used for research purposes. Participation by underage migrants (< 18 years) was only possible with additional information and the consent of their legal guardians. For this purpose, the consent form and study information were distributed to the students one week prior to the realization of the focus groups. This way, the underage students could take them home and bring the signed consent form back on the day of the focus group at the latest. Students without a signed consent form could not participate.

## Results

### Person(s) who perceived discrimination

The adolescents reported on experiences of discrimination that happened to them personally, to themselves in the company of other refugees/migrants, on experiences that they observed in other people and on experiences that were told to them by others (Table 1).

**Table 1:** Person(s) who perceived discrimination

Subcategories	Example
Self-experienced	*“Bus drivers always look at me crossly. It's like I’m driving with a bomb. […] Once I wanted to buy a bus ticket. Then the bus driver said, don’t come closer”* (FG 2)
Self-experienced in the company of other refugees/migrants	*“A woman approached us and asked why you don’t speak German. We are in Germany.”* (FG 2) (situation that happened to a student and her friend in a public bus)
Observed experiences	*“My father worked in the garden and she [the neighbor] said work sets you free”* (slogan is known for appearing on the entrance of Auschwitz and other Nazi concentration camps; FG 2)
Experiences heard about	*“I know people who can’t get a flat because they are foreigners “* (FG 1)

*FG* focus group

### Discriminating person(s)

We identified 56 codes concerning persons or institutions from whom the participants perceived discrimination and grouped them into nine subcategories (Table 2).

**Table 2:** Person(s)/Institution(s) from whom discrimination was perceived

Subcategories	*n*	Example
Random encounters	20	„*Old people* “ on the street (FG 2)
People in authority	16	Teachers, employers, coaches
Fellow students/players (sport)	5	“Once we played football […] and won and then they [opponents] said `F***ing foreigners, why don’t you go back to your home country?`” (FG 1)
Laws/Policy	5	No work permit in case of toleration/temporary visa
Service providers/customers	3	Bus driver, cashier at the supermarket
Flat owners/landlords	3	*„They [landlords] said: ‚You are foreigners, you get money from the city and so, that's why we don’t want to give you the flat.‘”* (FG 2)
Neighbors	2	*“…some neighbors don’t say hello back…”* (FG 3)
Other foreigners	1	*“For example, other foreigners, when we make mistakes, or we don’t say the right [German] word, they laugh.”* (FG 3)
Politically motivated people	1	Demonstrators

*FG* focus group

### Forms of discrimination

In our transcripts, we coded 90 cases of perceived discrimination and summarized them to 19 subcategories. According to the students, the form of discriminatory behavior they experienced the most was lack of understanding/lack of intercultural competence/prejudices. The second most common type of discrimination reported by the migrant and refugee students was (social) exclusion. Just as often they experienced discrimination based on laws and in the form of verbal insults. At school in particular, the students reported situations in which they felt unequally treated by teachers compared to German students. Other subcategories that could be formed from the students' experiences were: rudeness, paternalism/no freedom of choice for school career, withholding something, punishments, evil looks, intimidation/ threats, devaluation, rejection, suspicion, provocation, laughing, exploitation, a racist demonstration and even one case of attempted arson. The formed categories and their examples are displayed in (Table 3).

**Table 3:** Experienced forms of discrimination

Subcategories	*n*	Example
Lack of understanding/ lack of intercultural competence/ prejudices	14	*„Some teachers want you to speak German as well as the Germans. That you can do everything, as if German was your mother tongue.“* (FG 1)
(Social) exclusion	8	*„I was in a German class at secondary school. […] The Germans didn't want to talk to me and I sat alone and did everything on my own.“* (FG 3)
Discrimination based on laws	8	*„[I want to work…] but I am not allowed because I do not have a residence permit.“* (FG 4)
Verbal insults	8	*„I wanted to go shopping. All of a sudden, a woman came and spoke to me. She said, ‚Where are you from? ‘ So I answered her. ‚What is your religion? ‘ I said, ‚Muslim’ Then she insulted me “* (FG 1)
Unequally treated by teachers compared to German students	7	*“One teacher is stricter with us than with the Germans. Once she was very angry and said, ‚If you don ‘t learn German well, one day you'll have to cut vegetables [as a job].‘“* (FG 3)
Rudeness	6	*„Some clients at work immediately become rude when they notice that I am a foreigner.“* (FG 3)
Paternalism/no freedom of choice for school career	4	*“I wanted to study, but was pushed [by the teachers] to do an apprenticeship”* (FG 5)
Withholding something	3	*“We didn't get a flat when we were looking for a flat and they said: "You are foreigners, you get money from the city, that's why we don't want to give you the flat.”* (FG2)
Punishments	3	*“Some teachers think we are deliberately doing something against them. And we get punished for that.”* (FG 3)
Evil looks	3	*“The old people look at you so crossly”* (FG2)
Intimidation/ threats	3	*“Someone on the street said: «This is not your country. You can't get a job here.»”* (FG 4)
Devaluation	3	*“Then they said that foreigners aren’t good in playing football.”* (FG 5)
Rejection	2	*“I was not allowed to play in a sports game because the coach said that the German could play better.”* (FG 5)
Suspicion	2	*“Cashier thought that his credit card was stolen.”* (FG 2)
Provocation	2	*“A man flicked a cigarette at me and said: «Foreigner, you must go to Syria.»”* (FG 4)
Laughing	1	*“The others laugh when a student makes a mistake or doesn't say the right German word.”* (F 3)
Exploitation	1	*“Refugees do not know [German] laws and their rights and are exploited by employers”* (FG 1)
Racist demonstration	1	*“They shouted (at demonstrations): «Disaster refugees out!»”* (FG 1)
Attempted arson	1	*„People tried to burn down our shelter for refugees “* (FG 1)

*FG* focus group

### Locations where discrimination has taken place

The students mentioned 18 places where they had experienced or witnessed discrimination. These places could be combined into six subcategories (Table 4). Students reported being discriminated against in public places such as offices, public transports or on the street, at school in the classroom or on the schoolyard, in their leisure time at sports clubs or swimming pools, in the context of work or even in their neighborhoods.

**Table 4:** Locations where discrimination was experienced or witnessed by the study participants

Subcategories	*n*	Example
In public	13	Offices, shopping, public transport, on the street
In the health system	11	Hospital, having no health insurance
At school (classroom, schoolyard)	9	*„[At school] Germans don't have detention if they are late or forget homework, we do.“* (FG 1)
At work/looking for a job/ looking for a training place/ in an internship	8	*„[…] refugees do not know the laws and their rights and are exploited by employers (e.g., get no holidays)“* (FG 1)
When looking for accommodation/at home/in the neighborhood	6	*"[We were searching for an apartment] and my father said, ‚I have four children.‘ And the landlady said, ‚better if you had four dogs or something, then you could move in.‘"* (FG 2)
In leisure time (Public swimming pool, sports club)	4	*„For example, when we were in the football team for the first time, they said foreigners can't play so well. They [German players] train first, then they play with us.“* (FG 5)

*FG* focus group

### Reactions of the person(s) affected to the discrimination

The reactions students or their acquaintances showed as response to perceived discrimination can be summarized in 12 subcategories that are divided into two main categories: actions and emotions/affects (presented in Table 5). The action the most mentioned was „leaving the situation “, followed by verbal defences, boycott/deception/resignation, physical violence and also showing no reaction at all.

**Table 5:** Adolescent’s reactions to the discrimination (divided into the main categories “actions” and “emotions/affects”)

Actions	*n*	Example
Leaving the situation	9	*“The coach didn’t let me play[…], so I left the sports club”* (FG 5)
Verbal self-defence	5	*“That made me really angry. And then I turned around and said to [the woman in the bus], "Are you kidding me or what?"* (FG 5)
Boycott/deception/resignation	3	*“I deliberately did badly on the entrance test so that I wouldn't have to get into the school I was supposed to go to but didn't want to”* (FG 5)
Physical violence	3	*"[…] then we caused stress, hit the others and stuff"* (FG 4)
No reaction	2	*“I didn't do anything. I went home.”*(FG 1)

Emotions/affects	*n*	Example
Anger/aggressiveness	7	*"[…] if someone insults me, of course I'm angry."* (FG 3)
Fear	4	*“I took my bus ticket and left. […]I got scared.”* (FG 1)
Unpleasant feeling/nervousness	3	*“You don't say anything. One becomes nervous.”* (FG 3)
Acceptance/understanding	2	*“I understood why she said that.”*(FG 3)
Resignation/demotivation	2	*“The teacher demotivates us, we are afraid of her.”* (FG 3)
Helplessness	1	*“I didn’t know what to do”* (FG 2)
Astonishment	1	*“I was astonished to see what happened.”* (FG 3)

*FG* focus group

In addition to the named behaviors, the students also reported various emotions they felt in relation to the experiences of discrimination. The named emotions/affects were anger/aggressiveness, fear, unpleasant feelings or nervousness, acceptance, resignation, helplessness and astonishment.

### Reasons for discrimination as assumed by the students

When asked about assumed reasons for their experiences of discrimination, the students named 46 reasons that we classified into eight categories (Table 6). Mostly they blamed the laws and their status as foreigners for their experiences. Their appearance, religion, and language would also play a big part. However, they noted that Germans seem to have prejudices that lead to discriminatory behavior.

**Table 6:** Reasons for discrimination as assumed by the adolescents

Subcategories	*n*	Example
Status as refugee or foreigner (Laws)	17	*“They would have given me a job, but they couldn’t because I don’t have a work permit”* (FG 5)
Religion	7	*“I went to the hospital once and did an internship as a nurse. I was there the whole week and on Friday they said you can't stay here anymore because of your headscarf.”* (FG5)
Appearance	6	Black hair, dark skin *„The old people look mean […] when you have black hair. […] They think you want to steal something “* (FG 2)
Language	5	Lack of German language skills *“In job interviews they think: ‘You can't speak German. You can't be hired because of the language’”* (FG 1)
Prejudices	5	*"I don’t get a job because people say that foreigners don't do anything [are lazy]."* (FG 5)
Preparation for life in Germany	3	*"Teachers [are so strict because they] want us to learn German rules."* (FG 3)
Fear	2	*"The bus driver looked like I had a bomb, he was scared".* (FG 2)
Anger	1	*"They [football opponents] insulted us because we won, they were mad"* (FG 1)

*FG* focus group

## Discussion

The aim of the present study was to explore the experiences of discrimination reported by migrant adolescents in a vocational school in Germany. In the conducted focus groups, we examined which persons were involved in the concrete situations, where and how the discrimination took place, which behavioral and emotional reactions the affected persons showed to the experiences and which reasons they assume for the discrimination. The explicit aim was to record the students' subjectively perceived experiences of discrimination. Objective criteria of discrimination were not taken into account. The following conclusions could be drawn from the interviews.

### Both the students themselves and their acquaintances perceive discrimination in Germany

Most participants reported at least one discriminatory experience that either they themselves or someone they know had experienced in Germany. Together with the results of representative surveys of migrants [36], this can be seen as indicator of the high risk of discrimination to which migrants are exposed in Germany. Accordingly, in a Germany-wide, representative survey, slightly more than half of the migrants stated that they have already experienced discrimination due to their origin. Among them, a quarter of the interviewees reported having experienced discrimination frequently [36]. In a study of our research team among Syrian refugees in Germany more than one-third of the participants perceived discrimination at least in low frequency [27].

### Students feel discriminated against by random encounters and people in authority

In our focus groups, most of the reported discrimination that was experienced by the study participants was caused by random encounters on the street or other public places. However, the students also reported numerous forms of unfair treatment by authority figures such as teachers or (potential) employers. Especially when it comes to job search, studies have already shown that applicants with a migration background are disadvantaged by employers. This seems to be especially the case for people from African, Arabic and Muslim countries of origin [37, 38].

### Migrant adolescents experience many different forms of discrimination: from prejudices and unfriendly behavior to the denial of benefits to verbal hostility

It became clear that people with a migration background in Germany are exposed to very different forms of discrimination. Most of the verbal and non-verbal discrimination reported was a lack of understanding, social exclusion, insults, devaluations, derogatory looks or being ignored. On the institutional level, students reported numerous experiences of discrimination by law. These included being denied a work permit or health insurance. Even if these cases of discrimination are legally permissible or explicitly required by law—for example in the case of access to the labor market [38]—these forms of discrimination were among the most burdensome for the young people. Students also reported on institutional discrimination in the school context. In line with this result, explanations that see the German school system itself as a decisive reason for migrants’ poorer school performance are on the rise: Institutional discrimination is assumed, which, for example, makes it difficult for migrants to receive recommendations for a higher qualified school career [39]. A paper examining the structural reasons for educational failures of Turkish youth in Germany concludes that the nature of the education system in Germany remains deeply “unequal,” “hierarchical” and “exclusive” [40]. This study also demonstrates that the country of origin or the immigrants' background is still a barrier to having access to education and the labor market of Germany [40]. Evidence supporting all these findings was also found in our focus groups.

On an individual level at school, our participants sometimes felt unequally treated by their teachers compared to German vocational students. Here, however, it is important to discuss to what extent the students had insights into the treatment of German students, since the classes are held separately and with different school concepts. Regardless of whether discrimination is actually taking place or is only subjectively perceived as such by the students, efforts can be made to improve the cultural awareness of teachers and other school personnel through dedicated training and culturally responsive teaching [41]. Previous research indicates that students achieve at higher rates when their teachers are culturally sensitive, utilize culturally relevant teaching methods, and can create a genuinely empathetic relationship with youth who are culturally different [41].

The most alarming racist act reported was an arson attack on the refugee shelter of one of the participants. According to statistics from the German Federal Criminal Police Office, several hundred attacks have been carried out on refugee homes in Germany since the beginning of 2015 [42]. In the case of refugees coming from regions of crisis and war, these traumatic experiences are particularly worrying, especially in combination with pre-migratory traumatisation that can lead to an increased risk of mental disorders, especially PTSD [43]. Migrant students feel discriminated against especially because of their ethnic origin.

The findings of our focus groups emphasize that the students attribute a large part of their experiences of discrimination to their origin and status as foreigners and refugees. Thus, the interviewees reported that discrimination often took place because of a characteristic that classifies them as foreigners (e.g., race, appearance, religion). This is in line with previous research about assumed reasons for discrimination [44]. According to an analysis of representative data published in 2018, people whose migration background is outwardly recognizable—for example through skin color—feel discriminated against more often (48%) than people without a “visible” migration background (17%) [44]. People who have an accent in addition to a recognizable migration background are particularly affected: In this case, 59% said they already had experienced discrimination [44].

### Migrant students frequently report discrimination in public spaces, at school, at work and in the housing market

If we look at the areas of life in which our study sample experienced discrimination, it turns out that it mainly took place in public, at school, in health care, in working life, and in the housing market. In our focus groups, discrimination was most frequently reported in public when accessing goods or services, when looking for a job, in the housing market and in the neighborhoods. However, some students also experienced discrimination in leisure activities, such as sports clubs. In comparison, figures from the Anti-Discrimination Agency in Germany show that complaints of ethnic discrimination are mostly received due to experiences of everyday racism in working life and in access to goods and services [45]. Unlike other social subsystems, discrimination in the housing market has been clearly demonstrated in the past. It is expressed by significant price discrimination as well as by increased difficulties in finding housing, which could be proven by different experiments [46, 47].

### Experiences of discrimination lead to fear and anger

The focus groups highlight that the experiences of discrimination had a great impact on the students’ well-being and behavior. The interviewees reported feelings of fear, nervousness, helplessness, and aggression following the discrimination. In addition to verbal defences and boycott actions, there were also physical confrontations in rare cases. However, a large proportion of respondents reported leaving the situation of discrimination to avoid confrontations. According to earlier research findings, all these reactions to discrimination seem to be typical and have already been found in several settings and populations [19, 48]. Since previous studies, having examined the association between discrimination and mental health, have found significant correlations between these constructs [5, 48], it must be assumed that discrimination not only has a short-term effect on the mood of those affected, but also can have an impact on their general mental health and well-being [5, 48]. According to a current review, (racial) discrimination appears to be a key acculturative stress factor for adolescents, has effects on their mental and physical health and is associated with lower self-esteem and lower well-being [49]. Further findings of that review link perceived or experienced discrimination of immigrant children and adolescents to other developmental outcomes, e.g., a low ability to conduct for interpersonal relationships, misconduct or delinquency, a lower sense of mastery and control, higher substance use, as well as lower life satisfaction and sense of competence [49]. The relation of discrimination to greater physiological stress, depressive symptoms, and disruptive school behavior has also been shown in a German study among immigrant youth [50]. Benner and colleagues’ [51] meta-analysis summarizes that experiencing ethnic discrimination poses adjustment threats socioemotionally (e.g., poor mental health), relationally and academically (e.g., negative behavior and poor academic achievement) [51]. So, as we can see from these results, the short-term reactions reported by our participants can also lead to long-term psychological complaints and need to be further investigated in future studies.

Strengths and limitations

Compared to previous papers, this study stands out due to its detailed analysis of the different aspects that play a role in the migrant adolescent’s perception of discrimination. Another positive aspect is the cooperation with the vocational school where the focus groups were conducted. On the one hand, this allowed access to a group that is difficult to recruit for study participations [52] and on the other hand, it allowed us to present our findings to the school and discuss possible improvements with the teachers. Although this study is one of the first qualitative studies to address the perceived discrimination of young people with a migration background in Germany, some limitations must be admitted. When evaluating the results, it should be noted that the focus groups were conducted in German. Since some of the students did not have sufficient knowledge of German at the time of the focus groups, it is possible that certain aspects were misunderstood and other important aspects were not mentioned. In addition, the focus groups were conducted during their school lessons. Therefore, it is conceivable that some students did not want to open up in front of their classmates and therefore did not report their experiences of discrimination. Although we did not have the impression that the sincerity and openness of the participants was impaired during the focus groups, a neutral interview setting would probably have increased the openness of the students.

Due to the exploratory and qualitative nature of the study, the results cannot be generalized to the entire community of migrant and refugee adolescents in Germany.

It also needs to be discussed to what extent the fact that the school is very committed to combating racism (“School against Racism”) had an influence on the results. Furthermore, the migrants and refugees in our sample are taught in their own classes, separate from the German students with little contact between the groups. In schools where these aspects are not the case and the migrants have more contact with the Germans, the results might be considerably different. Therefore, our results require replication in larger and more representative samples. Moreover, future studies should investigate further aspects and effects of discrimination.

## Conclusions

Discrimination is one of the most substantial barriers for successful adaptation and psychological functioning of migrant adolescents. Combating it may once again be particularly relevant in the current refugee movement from Ukraine. There is strong empirical evidence that perceived discrimination is related to lower overall well-being and worse mental health (e.g., [5, 9]). Understanding where and how perceptions of discrimination arise is crucial for the German society that is faced with the task of successfully integrating thousands of immigrants.

The adolescents in our focus groups and their acquaintances (families and friends) experienced discrimination in Germany in several forms, places and by a variety of people and laws. As the first step on the way to preventing discrimination, we suggest the sharing of culturally sensitive information on this issue. On the one hand, it is urgently necessary to inform refugees about their right to non-discrimination and public support services. On the other hand, with a view to effectively combating discrimination, not only should those affected and their support structures be strengthened, but also the perpetrators of discrimination must be targeted. Often, the discriminators are not aware that their behavior leads to a perception of discrimination on the part of their counterparts. At this point, culturally sensitive training must take place, especially in schools.

## Data Availability

The dataset analyzed during the current study is available from the corresponding author on reasonable request.
